# Convergent weaponry in a biological arms race

**DOI:** 10.7554/eLife.08710

**Published:** 2015-06-11

**Authors:** Edward N Baker, Paul G Young

**Affiliations:** Maurice Wilkins Centre for Molecular Discovery and the School of Biological Sciences, University of Auckland, Auckland, New Zealanden.baker@auckland.ac.nz; Structural Biology Group, School of Biological Sciences, University of Auckland, Auckland, New Zealand

**Keywords:** *Streptococcus pyogenes*, *Streptococcus pneumoniae*, *Clostridium perfringens*, host-microbe interactions, fibrinogen, bacterial surface protein, other

## Abstract

Bacterial surface proteins covalently attach to host cells via a mechanism that is also used by immune system proteins that help eliminate invading pathogens.

**Related research article** Walden M, Edwards JM, Dziewulska AM, Bergmann R, Saalbach G, Kan SY, Miller OK, Weckener M, Jackson RJ, Shirran SL, Botting CH, Florence GJ, Rohde M, Banfield MJ, Schwarz-Linek U. 2015. An internal thioester in a pathogen surface protein mediates covalent host binding. *eLife* **4**:e06638. doi: 10.7554/eLife.06638

**Image** Hundreds of proteins from the surfaces of bacterial cells are predicted to contain reactive thioester bonds

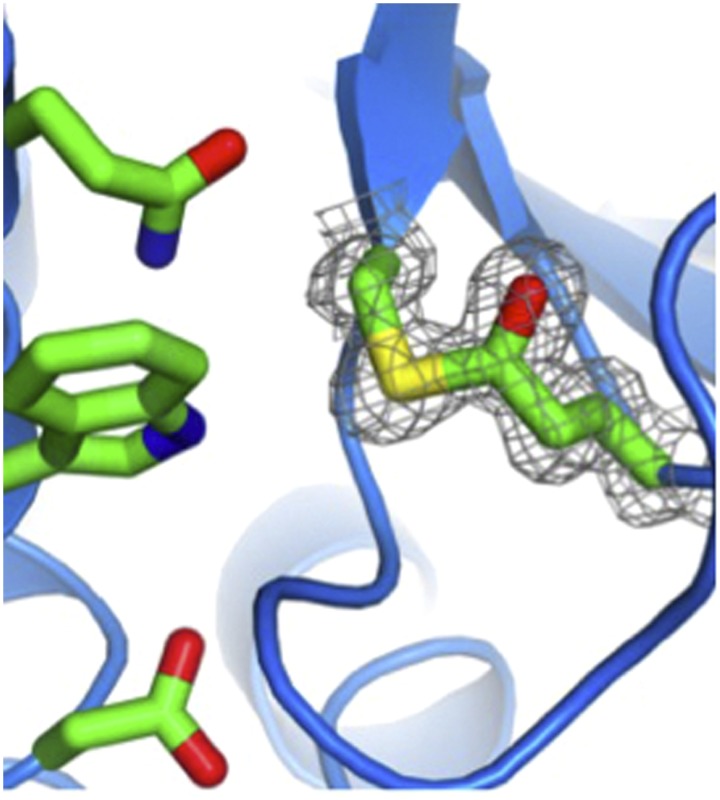

Proteins carry out a vast variety of biological processes, usually with an exquisite specificity that is determined by the three-dimensional structure of each protein. The amino acids that make up proteins have chemically diverse side chains, but the side chains within a protein rarely react with one another. Evolution has evidently selected for protein structures that keep reactive side chains apart, or limit their reactivity—probably to reduce the potential for proteins to fold into the wrong shapes.

Until recently it was thought that disulfide bonds were the only covalent bonds that commonly formed between the side chains within a protein, but recent analyses of bacterial genomes have revealed a goldmine of previously unknown covalent bonds within proteins. Curiously, all of these ‘intramolecular’ covalent bonds have been found in proteins that are displayed on the surface of Gram-positive bacteria. These bonds include isopeptide bonds (Kang et al., 2007) and ester bonds (Kwon et al., 2014), both of which act to stabilise the proteins in filaments (called pili) that bacteria use to attach themselves to surfaces.

A third, and equally unexpected, discovery was the presence of thioester bonds between cysteine and glutamine side chains in a protein called Cpa. This protein is the adhesive subunit that is found at the tips of the pili made by a bacterium called *Streptococcus pyogenes* (Pointon et al., 2010; Linke-Winnebeek et al., 2014). This bacterium is a common cause of throat infections in humans, but it can also cause more serious, and sometimes life-threatening, infections. Similar thioester bonds were already known as the highly reactive ‘warheads’ of the complement proteins that are involved in the human immune system. As such, the obvious, but unproven, implication was that *S. pyogenes* had evolved similar weaponry to attack its human host.

Now, in *eLife*, Uli Schwarz-Linek from the University of St. Andrews, Mark Banfield from the John Innes Centre and colleagues—including Miriam Walden and John Edwards as joint first authors—report a comprehensive analysis of bacterial proteins that contain thioester bonds (Walden et al., 2015). In addition to demonstrating that such proteins are common in Gram-positive bacterial pathogens, they also dig deeper and identify a molecular target of one of these proteins.

Walden, Edwards et al. first searched bacterial genomes and found similar protein sequences that contained conserved cysteine and glutamine residues in hundreds of cell-surface proteins from Gram-positive microorganisms. Next, they expressed 12 of these putative thioester-containing protein domains (which they called TEDs for short) and solved the three-dimensional structures for four of them using X-ray crystallography. They then used mass spectrometry to confirm that the proteins contained thioester bonds, as predicted; the bonds could also be seen in the crystal structures.

Thioester bonds are known to be highly reactive (Law and Dodds, 1997). However, finding an exact molecular target for a TED was much tougher; indeed, this challenge has still not been accomplished for the much more intensively studied complement proteins. For this purpose, Walden, Edwards et al. decided to focus on SfbI—a TED-containing protein from *S. pyogenes*—because it had been reported to bind to the human protein fibrinogen.

Best known as a component of blood plasma that is essential for blood clotting, fibrinogen is also found in the extracellular matrix that supports cells, and on the surface of inflamed cells. Walden, Edwards et al. found that SfbI could irreversibly bind to isolated fibrinogen, and even pull fibrinogen out of a mixture of proteins found in blood plasma. Both of these activities depended on SfbI's thioester bond, and importantly, no other proteins were pulled down from the blood plasma.

Mass spectrometry and chemical labelling then established that a single amino acid—a lysine referred to as Lys100 in the Aα chain of fibrinogen—is the sole target of the thioester bond in SfbI (Figure 1). Fibrinogen is converted into long fibres of fibrin during blood clotting, and bacterial cells (*Lactococcus lactis*) that were engineered to express SfbI also bound to fibrin via the reactive thioester bond. Finally, Walden, Edwards et al. turned to whole-cell experiments, using a human cell line that mimics inflammation. SfbI was found to bind to these cells, via the fibrinogen on their surfaces: again this depended on the thioester bond in SfbI.

**Figure 1.**
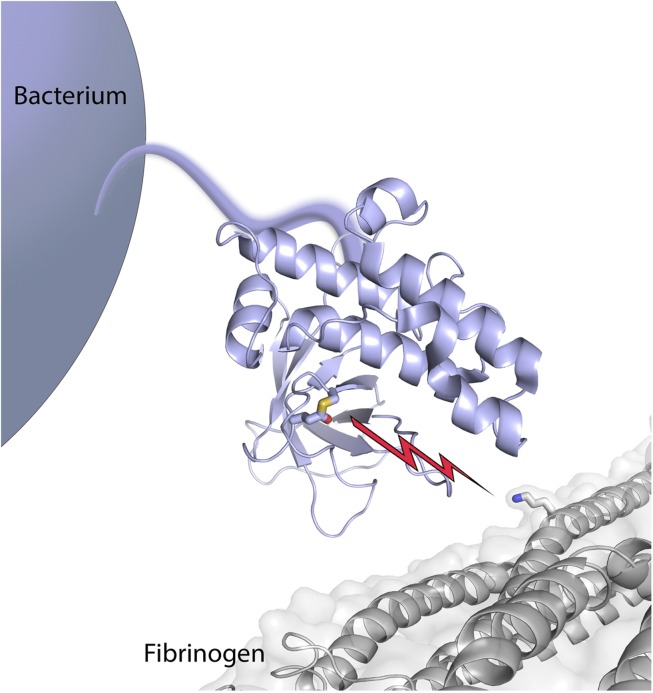
Bacterial surface protein SfbI poised to attack its target, fibrinogen. The thioester-containing domain (lilac) of SfbI is anchored to the cell wall of a *Streptococcus pyogenes* bacterium by an extended stalk. Walden, Edwards et al. report that the reactive thioester bond (shown as sticks, with the sulfur atom from the cysteine coloured in yellow, and the oxygen atom from the glutamine in red) attacks its target, fibrinogen (coloured grey); it then forms a new covalent bond (depicted by the lightning bolt) with the side chain of a lysine in fibrinogen (also shown in stick mode, with the nitrogen atom in blue).

What is fundamentally new is the demonstration that these thioester-containing proteins bind their targets through covalent chemical bonds. This is in complete contrast to other known methods of adhesion, which typically rely on the combined strength of many weaker non-covalent interactions. The cysteine–glutamine thioester bond breaks, and a new bond then forms between the glutamine and its target—a reaction that Walden, Edwards et al. liken to the firing of a ‘chemical harpoon’. The molecules targeted by TEDs are likely to vary, since the protein sequences of most TEDs share very little in common. Indeed, of the TEDs tested so far, only SfbI bound specifically to fibrinogen. But the fundamental principle uncovered here seems widespread.

From a protein point of view, one of the most intriguing insights from this work concerns the relationship between the bacterial TEDs and the complement family. Complement proteins are thought to react with the surface of pathogens, and irreversibly tag them for elimination by the host's immune system. These proteins (and related protease inhibitors) are evolutionarily ancient, but are the only known proteins with integral thioester bonds (Levashina et al., 2001). In these proteins, the thioester is usually buried inside the structure, and is only activated when a large conformational change exposes it (Janssen et al., 2006). The bacterial TEDs, in contrast, have a completely different shape and no such control mechanism. Instead, the cysteine–glutamine bond is partially covered by adjacent loops of the protein chain that likely limit its reactivity.

It seems clear that these two systems have evolved independently in eukaryotic and bacterial species, in a beautiful example of convergent evolution. How ironic that a weapon used by humans to attack and eliminate pathogens should also be used by those same pathogens to attack us!
